# Relationship between Personality and Gray Matter Volume in Healthy Young Adults: A Voxel-Based Morphometric Study

**DOI:** 10.1371/journal.pone.0088763

**Published:** 2014-02-14

**Authors:** Fengmei Lu, Yajun Huo, Meiling Li, Heng Chen, Feng Liu, Yifeng Wang, Zhiliang Long, Xujun Duan, Jiang Zhang, Ling Zeng, Huafu Chen

**Affiliations:** Key Laboratory for Neuroinformation of Ministry of Education, School of Life Science and Technology, University of Electronic Science and Technology of China, Chengdu, Sichuan, People’s Republic of China; Centre Hospitalier Universitaire Vaudois Lausanne - CHUV, UNIL, Switzerland

## Abstract

This study aims to investigate the neurostructural foundations of the human personality in young adults. High-resolution structural T1-weighted MR images of 71 healthy young individuals were processed using voxel-based morphometric (VBM) approach. Multiple regression analyses were performed to identify the associations between personality traits and gray matter volume (GMV). The Eysenck Personality Questionnaire-Revised, Short Scale for Chinese was chosen to assess the personality traits. This scale includes four dimensions, namely, extraversion, neuroticism, psychoticism, and lie. Particularly, we studied on two dimensions (extraversion and neuroticism) of Eysenck’s personality. Our results showed that extraversion was negatively correlated with GMV of the bilateral amygdala, the bilateral parahippocampal gyrus, the right middle temporal gyrus, and the left superior frontal gyrus, all of which are involved in emotional and social cognitive processes. These results might suggest an association between extraversion and affective processing. In addition, a positive correlation was detected between neuroticism and GMV of the right cerebellum, a key brain region for negative affect coordination. Meanwhile, a negative association was revealed between GMV of the left superior frontal gyrus and neuroticism. These results may prove that neuroticism is related to several brain regions involved in regulating negative emotions. Based on those findings, we concluded that brain regions involved in social cognition and affective process accounted for modulation and shaping of personality traits among young individuals. Results of this study may serve as a basis for elucidating the anatomical factors of personality.

## Introduction

Extraversion and neuroticism are two important and frequently studied dimensions of human personality [1], [2]. According to Eysenck’s influential arousal theory [2], [3], extraversion is the result of individual differences in the level of activity in the cortico-reticular loop and other arousal systems (e.g., monoamine oxidase system and pituitary-adrenocortical system). Thus, individuals with low extraversion scores have higher baseline levels of cortical arousal and higher arousability of the cortex, i.e., greater changes in cortical activity in response to arousing stimuli, than individuals whose extraversion scores were high [4], [5]. Neuroticism, on the other hand, results from a lower threshold for activation in the limbic circuit, such that individuals with high neuroticism scores show great activation levels and low thresholds within subcortical structures [1], [2].

The aforementioned biological explanations of extraversion and neuroticism have attracted increasing interests in linking the two fundamental traits to functional and anatomical brain markers. On the functional level, many neuroimaging studies have examined extraversion and neuroticism using modern neuroimaging techniques, such as positron emission tomography, electroencephalogram, magnetic resonance perfusion imaging, and resting-state functional magnetic resonance imaging (fMRI) [6]–[11]. These studies have highlighted the functional neural correlations of extraversion and neuroticism, and further demonstrated the underlying neural mechanisms of personality dimensions, thereby providing neurobiological evidence for the hypothesized biological model of Eysenck’s personality.

Recent studies have used modern structural imaging techniques to detect whether or not personality traits are correlated with brain structures. Previous studies have suggested that personality may be associated with brain regions involved in emotional regulation and affective processing. For example, in an adolescent sample, Blankstein et al. [12] found that extraversion and neuroticism were negatively and positively correlated with the GMV of the medial frontal gyrus and subgenual anterior cingulate cortex in females, respectively. The opposite of this relationship between GMV and personality could be observed in males. Omura et al. [13] studied forty-one participants aged 19 to 29 years and found that extraversion was positively correlated with the left amygdala and orbitofrontal cortex but negatively correlated with the precentral cortex. DeYoung et al. [14] studied individuals aged 18 to 40 years and found that extraversion was positively correlated with GMV of the orbitofrontal cortex. Another VBM study [15] involving sixty-five participants aged 21 to 56 years revealed that extraversion was positively correlated with the anterior cingulate cortex volume, orbitofrontal volume, and right amygdala volume. This study also suggested that the correlation between extraversion and the volume of the anterior cingulate cortex varied in males and females. Obviously, the main results of these studies are inconsistent. Age effect is a dominant factor affecting changes in regional brain volume, and personality may interact with the effects of age on brain structures [16]. This inconsistency may be due in part to the age range of the recruited subjects [16]. To remove the potential effect of the wide age range, we concentrated on examining the relationships between personality traits and GMV of brain regions only in young adults.

Our aims in the present study were two-fold: first, we examined whether there are relationships between extraversion and neuroticism based on Eysenck’s model and regional brain GMV in young adults; second, we investigated whether the core brain structures involved in emotional processing are correlated with extraversion and neuroticism. Of note, since the definition and the neuropsychological mechanism of psychoticism, the third dimension in the Eysenck scale, are ambiguous [17], [18], we did not investigate the relationship between psychoticism and GMV.

## Materials and Methods

### Participants

Seventy-one right-handed healthy university students (34 males) underwent magnetic resonance imaging (MRI) scans at Jinling Hospital, Nanjing, China. The mean age of the participants was 22.35 years (standard deviation = 1.5, age range: 19–26 years). Informed written consents were obtained from all participants before any study procedure was initiated. Exclusion criteria included any history of psychiatric or neurological illness, brain injury, and alcohol or drug abuse. An informal interview prior to the MRI scanning was conducted to confirm that subjects did not use any psychotropic drugs. Left-handed individuals, who were assessed using Chinese revised version of the Edinburgh Handedness Inventory, were excluded [19]. The present study was approved by the local Medical Ethics Committee at Jinling Hospital, Nanjing University.

### Personality Questionnaires

To assess personality traits, all participants completed the Eysenck Personality Questionnaire-Revised, Short Scale for Chinese (EPQ-RSC), a self-report questionnaire [20]–[23]. We chose the EPQ-RSC since it has been extensively used for clinical and research purposes in China. Furthermore, the validity and stability of this scale have been authenticated in Chinese subjects. The EPQ-RSC includes four dimensions: Extraversion (E), Neuroticism (N), Psychoticism (P), and Lie (L). Subjects responded to each item in the questionnaire with “yes” or “no” (coded as 1 or 0) depending on the applicability of the statement. The raw scores were translated into *T*-scores using the following formula [22]:
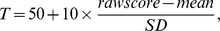
where *mean* is the mean value of the personality scores over all participants; *SD* is the standard deviation of the personality scores. Qian et al. [22] suggested that EPQ-RSC had satisfactory reliability and validity of extraversion, neuroticism and lie, whereas the reliability and validity of psychoticism were relatively lower. We focused our analyses on extraversion and neuroticism, two important and significant personality dimensions whose resultant *T*-scores were used for measuring correlations with GMV values in the present work.

### MRI Data Acquisition

High-resolution 3D structural MRI scans of all subjects were performed on a 3T MRI scanner (Siemens-Trio, Erlangen, German) using a T1-weighted spoiled grass gradient recalled sequence at the Jinling Hospital, Nanjing, China. Tight but comfortable foam padding was used to minimize head motion, and earplugs were used to reduce scanner noise. The following parameters were used: repetition time (TR) = 2300 ms, echo time (TE) = 2.98 ms, flip angle = 9°, slice thickness = 1 mm, FOV = 24 cm×24 cm, matrix size = 512×512, and voxel size = 0.5 mm×0.5 mm×1 mm.

### Data Preprocessing

Images were initially visually inspected for artifacts or structural abnormalities unrelated to healthy subjects. Subjects with general MRI contraindications were excluded in the following analyses. VBM analyses were performed using SPM8 (http://www.fil.ion.ucl.ac.uk/spm) as previously described [24]. The detailed procedures were as follows. First, the origin of each participant’s structural images was set to the anterior commissure manually. Second, all images were divided into gray matter, white matter, and cerebrospinal fluid, and then imported into a strictly aligned space [25]. Third, the segmented images were iteratively registered by the Diffeomorphic Anatomical Registration Through Exponentiated Lie Algebra toolbox [26]. This process created a template for a group of individuals. The resulting images were spatially normalized into the MNI space using an affine spatial normalization. An extra processing step was performed to multiply each spatially normalized image by its relative volume before and after normalization to maintain the total amount of each tissue. Finally, images were smoothed with an isotropic Gaussian kernel of 8 mm full width at half maximum.

### Second-level Analyses

Voxel-based multiple regression analyses (based on general linear model) were performed by SPM8, with voxel-wise GMV value as dependent variable and N and E scores of personality traits as covariates of interest. In addition, sex, age, and total intracranial volume were used as external regressors to control their effects on both brain structure [27], [28] and personality [29]. We set the significance value at *p*<0.05 using the AlphaSim correction (combined height threshold of *p*<0.005 and a minimum cluster size of 172 voxels). This correction was conducted using the AlphaSim program embedded into the REST Software (http://www.restfmri.net/forum/REST_V1.8), which applied Monte Carlo simulation to calculate the probability of false positive detection by considering both the individual voxel probability threshold and cluster size [30].

## Results

### Sample Characteristics and Personality Scores

The descriptive statistics of sample characteristics and personality scores are shown in **Table 1**. A significantly negative correlation was found between N and E (*r* = −0.4042, *p*<0.0001). The result was concordant with many previous studies, demonstrating an inverse correlation between neuroticism and extraversion [31]–[33].

**Table 1:** Descriptive statistics of sample characteristics and personality scores.

Variable	Data	Range
Age (years)	22.35±1.5	19–26
Gender (male/female)	34/37	–
E scores	54.68±8.39	35.75–73.05
N scores	44.42±11.95	24.98–65.70

Age and personality scores are displayed as *mean*±*SD*.

### Personalities and GMV Values

**Figure 1:**
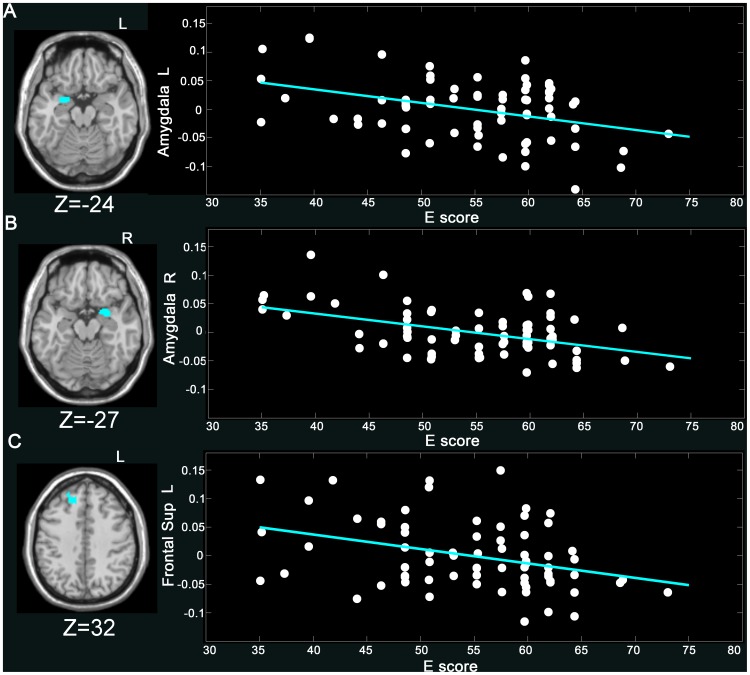
Negative correlation of E score and GMV (*p*<0.05, Alphasim corrected) in the (A) L amygdala (−27, 3, −24) (*r* = −0.43, *p*<0.001), (B) R amygdala (27, 2, −27) (*r* = −0.46, *p*<0.001), and (C) L superior frontal gyrus (−14, 29, 32) (*r* = −0.35, *p*<0.005). The GMV values in the figure were extracted from the significant clusters after age, gender, and total intracranial volume of each subject were regressed out. More details of these regions are described in **Table 2**.

E scores were negatively correlated with GMV of the left amygdala (coordinates: x = −27, y = 3, z = −24), the right amygdala (coordinates: x = 27, y = 2, z = −27), the left parahippocampal gyrus (coordinates: x = −18, y = 3, z = −24), the right parahippocampal gyrus (coordinates: x = 27, y = 6, z = −26), the right middle tem*p*oral gyrus (coordinates: x = 54, y = −71, z = 20), and the left superior frontal gyrus (coordinates: x = −14, y = 29, z = 32) (**Figures 1** **–** **2**, **and** **Table 2**).

**Figure 2:**
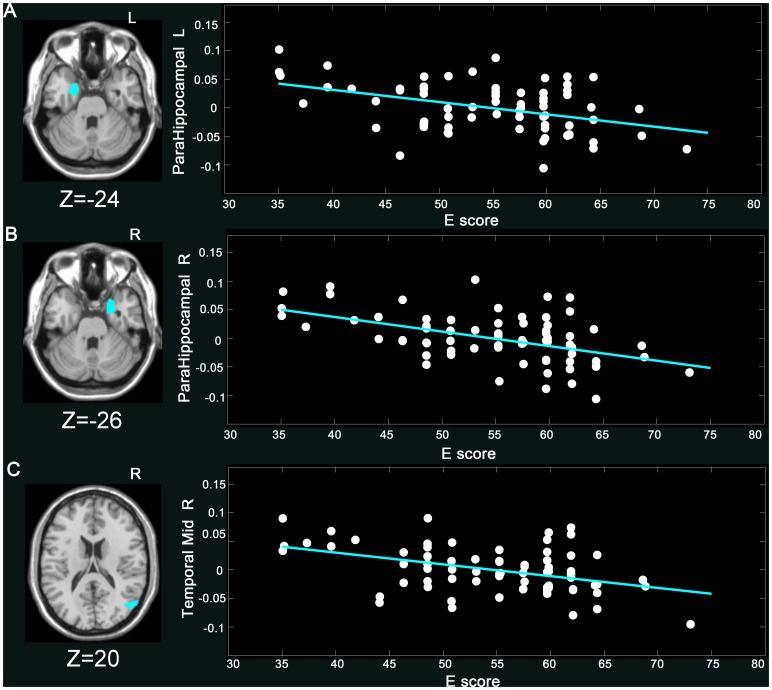
Negative correlation of E score and GMV (*p*<0.05, Alphasim corrected) in the (A) L parahippocampal (−18, 3, −24) (*r* = −0.42, *p*<0.0001), (B) R parahippocampal (27, 6, −26) (*r* = −0.50, *p*<0.0001), and (C) R middle temporal gyrus (54, −71, 20) (*r* = −0.38, *p*<0.05). The GMV values in the figure were extracted from the significant clusters after age, gender, and total intracranial volume of each subject were regressed out. More details of these regions are described in **Table 2**.

**Table 2:** Brain regions in which GMV were significantly related with E and N.

Personalities	Regions	BA	MNI coordinate			Direction of association	*T* value
			x	y	z		
E	L AMYG	–	−27	3	−24	Negative	−4.22
	R AMYG	–	27	2	−27		−5.11
	L ParaHG	34	−18	3	−24		−4.02
	R ParaHG	28	27	6	−26		−5.10
	R MTG	39	54	−71	20		−3.22
	L SFG	8/9/32	−14	29	32		−3.63
N	R CER	–	8	−41	−14	Positive	3.36
	L SFG	6/8	−20	13	56	Negative	−3.46

MNI, Montreal Neurological Institute; BA, Brodmann’s area; E, extraversion; N, neurocitism; L, Left; R, right; AMYG, amygdala; ParaHG, parahippocampal gyrus; MTG, middle temporal gyrus; SFG, superior frontal gyrus; CER, cerebellum. *T* value represents the statistical value of peak voxel showing brain regions’ GMV correlated with E and N. Positive and negative *T* values indicate positive and negative correlations, respectively, between GMV and E/N scores.

**Figure 3:**
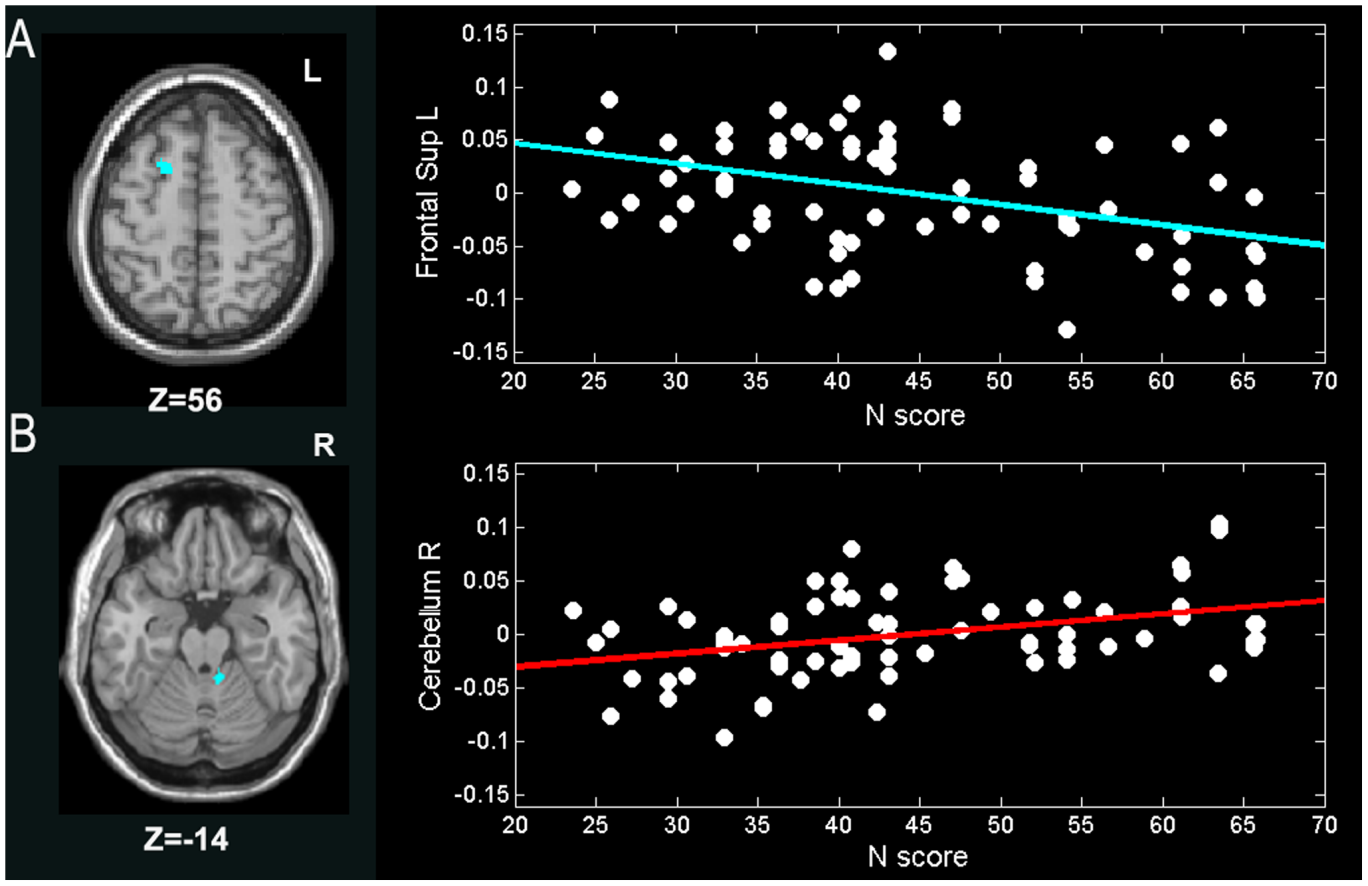
Correlations of N score and GMV (*p*<0.05, Alphasim corrected) in the (A) L superior frontal gyrus (−20, 13, 56) (*r* = −0.3853, *p*<0.0001), and (B) R cerebellum (8, −41, −14) (*r = *0.3720, *p*<0.05). The GMV values in the figure were extracted from the significant clusters after age, gender, and total intracranial volume of each subject were regressed out. More details of these regions are described in **Table 2**.

N scores were positively correlated with GMV of the right cerebellum (coordinates: x = 8, y = −41, z = −14) but negatively correlated with GMV of the left superior frontal gyrus (coordinates: x = −20, y = 13, z = 56) (**Figure 3** **and** **Table 2**).

The results of our analyses are shown in **Table 2**, which lists all clusters related to Eysenck’s personality of extraversion and neuroticism, controlling the effects of age, sex, and total intracranial volume.

## Discussion

In the current study, we investigated two dimensions (extraversion and neuroticism) of Eysenck’s personality in young people. Extraversion and neuroticism are personality traits linked to emotional states. In general, extraverted individuals are susceptible to positive emotions, such as sociability, excitement, engagement, and enthusiasm, whereas neurotics are particularly susceptible to negative emotions, including fear, anxiety, and distress [32]. Controlling for age, sex, and total intracranial volume, we explored the correlations between extraversion and neuroticism and GMV values. The GMV of the bilateral amygdala, the bilateral parahippocampal gyrus, and right middle temporal gyrus were negatively correlated with extraversion. The GMV of the left superior frontal gyrus was negatively associated with both extraversion and neuroticism, whereas that of the right cerebellum was positively correlated with neuroticism. These results help to clarify the important relationships between the GMV of brain regions associated with emotional processing and personality traits.

### GMV of Amygdala Negatively Associated with E

In the present study, the relationships between extraversion and GMV in several brain areas (the bilateral amygdala, the bilateral parahippocampal gyrus, the right middle temporal gyrus and the left superior frontal gyrus) were all found to be negative. This result indicates that individuals with high extraversion scores showed small GMV in the reported regions.

The direction of associations found in this study is consistent with previous structural studies that showed only negative correlations between extraversion and several frontal regions [31], [34], [35]. However, a positive relationship was also found between amygdala and extraversion in a biological model of five-factor traits [13], [36]–[38]. For example, Cremers et al. [38] observed that extraverts, compared with introverts, showed higher total GMV in the right amygdala. Omura et al. [13] discovered a positive association between extraversion and GMV in the left amygdala. Our inconsistent results may be partly attributed to many factors, including heterogeneity in personality measurements between the five-factor and Eysenck’s models. Some neurodevelopmental studies have reported that lower grey matter density can lead to better performance [39]–[42]. A smaller GMV may possibly indicate a higher efficiency in implementing specific functions associated with the amygdala. The association between extraversion and reduced amygdala GMV may have clinical significance since extraversion was found to be positively related to positive emotions after the induction of pleasant mood under Eysenck’s model [5]. Therefore, the relationship between GMV and extraversion in the amygdala, which is essential in emotional processing, supports Eysenck’s prediction for extraverts with positive effect.

### GMV of the Parahippocampal Gyrus Negatively Associated with E

We also found an association between reduced GMV in the parahippocampal gyrus and extraverts. Jackson et al. [16] concluded that the interactive effects of personality with age in the parahippocampal gyrus were related to its function in emotional and memory processing. Moreover, Mobbs et al. [43] used event-related functional MRI to address the putative neural and behavioral associations between humor appreciation and extraversion. They found a positive correlation between humor-related activation and extraversion in the parahippocampal gyrus. Extraversion, according to Eysenck’s theory, results from the differences between the activity levels of an individual in the cortico-reticular loop and other arousal systems (e.g., monoamine oxidase and pituitary-adrenocortical systems) [3], [44], [45]. Those previous results suggest that the parahippocampal gyrus contributes to emotional processing, which is an important aspect in modulating or shaping personality. Our findings may sustain the idea that activation in the parahippocampal gyrus is involved in the positive effect. The current result showing larger GMV in introverts is also in line with the result of the previous studies, in which individuals who exhibited lower E scores showed greater neuronal coherence in the parahippocampal gyrus [9], [10].

### GMV of the Middle Temporal Gyrus Negatively Associated with E

Negative correlation between extraversion and the GMV of the middle temporal gyrus was an additional interesting result. Previous studies demonstrated that the middle temporal gyrus is involved in several cognitive processes connected with personality traits [46]. Moreover, the middle temporal gyrus is involved in language and semantic memory processing [46]–[48], and multimodal sensory integration [49]. The relationship between GMV and extraversion detected in the right middle temporal gyrus suggested that extraverts are different from introverts in emotional processing. This suggestion was based on the assumption that extraverts are more behaviorally active, optimistic, and sociable than introverts [50], [51]. Lower grey matter density can lead to better performance [52]. Thus, we can conclude that extraverts perform working memory tasks more efficiently than introverts.

### GMV of the Superior Frontal Gyrus Negatively Associated with E and N

A significantly negative relation was observed in the left superior frontal gyrus between GMV and both extraversion and neuroticism. The superior frontal gyrus was thought to contribute to higher cognitive functions and particularly to working memory [53]. In fMRI experiments, Goldberg et al. [54], [55] found that the superior frontal gyrus was involved in self-awareness, in coordination with the action of the sensory system. Kunisato et al. [56] have explored positive correlations between extraversion and fractional amplitude low-frequency fluctuations in the right superior frontal gyrus. Forsman et al. [57] also observed a negative relationship between the GMV of the left superior frontal gyrus and extraversion. Neuroimaging results indicated that the left superior frontal gyrus was significantly correlated with the regulation of negative emotions and partially significantly activated in regulating positive emotions [58], [59]. Personality traits were conjunct with emotions, in that the superior frontal gyrus involved in both positive and negative effects was also involved in extraversion and neuroticism.

### GMV of Cerebellum Positively Associated with N

Our results showed that the GMV of the cerebellum was positively correlated with neuroticism. The cerebellum was involved in emotional processing, cognition, and regulation of mood [60]–[64]. Previous studies have demonstrated that the cerebellum was principally involved in motor and cognitive functions [65]–[69], less involved in emotional regulation and affective processing [70]–[72], and even less involved in personality individual differences [73]. A recent study in which cerebellar function was manipulated with transcranial direct current stimulation provided an additional evidence for cerebellar involvement during the processing of negatively valenced emotional stimuli [74]. Research has shown a positive relationship between neuroticism and high levels of the stress-related hormone cortisol [75]. Moreover, De Young et al. [14] found that the cerebellum was positively associated with neuroticism. Our results suggested that individuals with higher N scores had higher GMV in the cerebellum, which may agree with the theory on the involvement of the cerebellum in the vulnerability to experiencing negative effects and developing mood disorders [74], [75]. In conclusion, the GMV of the cerebellum is associated with neurotic personality traits, which are obviously related to negative emotions.

## Limitations

Several limitations of the current study should be mentioned. First, our study was limited by a relatively small sample size. An expanded imaging dataset will be necessary to confirm our preliminary results in the future study. In addition, our study was an exploratory research. Thus, the AlphaSim correction was adopted for the second-level analyses. In the future, more conservative correction (such as family wise error (FWE) or false discovery rate (FDR) correction) should be applied to avoid false positives. Third, we examined the relationship between GMV and personality traits only in young adults sample in our study, future studies should include young, middle-aged, and elderly adults simultaneously in order to directly explore the effects of age on the relationship between personality and GMV. Finally, the obtained results were based on univariate statistical method. Previous studies have suggested that multivariate method is sensitive to the fine-grained spatial discriminative patterns and outperforms univariate analysis [76]–[78]. Future studies may be benefit from a multivariate approach to explore the neurostructural foundations of the human personality.

## Conclusions

We used VBM, an objective structural analysis technique designed to evaluate brain structural features, to characterize the relationship between personality traits and GMV of brain regions in young adults. Extraversion was negatively correlated with GMV of the bilateral amygdala, the bilateral parahippocampal gyrus, the right middle temporal gyrus, and the left superior frontal gyrus. These results indicated that brain regions involved in the initial process of emotional information affected personality. In addition, a positive correlation was detected between neuroticism and GMV of the right cerebellum, whereas a negative association was determined between GMV of the left superior frontal gyrus and neuroticism. This result indicated that those regions contributed to negative emotional processing were associated with neuroticism. Overall, the present findings suggested that brain structures involved in social cognition and affective process accounted for modulation and shaping of personality in young people. We concluded that relationships do exist between GMV of brain regions and personality. Our conclusion was in accordance with the results of previous studies, which suggested that human personality traits are based on individual differences in brain structures.
